# 3D-Printed Mycelium Biocomposites: Method for 3D Printing and Growing Fungi-Based Composites

**DOI:** 10.1089/3dp.2023.0342

**Published:** 2025-04-14

**Authors:** Danli Luo, Junchao Yang, Nadya Peek

**Affiliations:** Human-Centered Design and Engineering, University of Washington, Seattle, Washington, USA.

**Keywords:** digital fabrication, open-source hardware, mycelium, 3D printing, compostable materials

## Abstract

Despite recent advances in 3D printing and additive manufacturing, the main materials in rapid prototyping are derived from finite resources such as petroleum-based plastics. Researchers are developing alternatives to exhaustible and potentially environmentally harmful materials through biomaterials. Mycelium biocomposites are one promising area of inquiry; when mycelium decomposes biomass, it produces a composite biomaterial, which is fully compostable and has beneficial structural and hydrophobic properties. However, mold-based fabrication methods for biocomposites require tooling and limit the possible shapes. We introduce a novel method for directly 3D printing mycelium biocomposites without the need for molds or tooling. Our method comprises three main contributions: Mycofluid, a mycelium-inoculated paste that uses spent coffee grounds, a recycled biomass; Fungibot, a custom hardware system for 3D printing biopastes like Mycofluid; and a method for incubating mycelial growth within fresh 3D prints resulting in mycelium biocomposite parts. We illustrate our contributions through a series of objects showcasing our method and the material qualities of the parts. Notably, we demonstrate how living mycelium can fuse separate prints, enabling complex geometries that are otherwise challenging to 3D print as one part.

## Introduction

Research in 3D printing and rapid prototyping is increasingly grappling with the waste generated in our own design, prototyping, and fabrication practices,^1^ an extension of concerns around the environmental impact from the manufacture, use, and disposal of commercial products.^2^ Responding to such concerns, researchers and designers have developed sustainably sourced materials and sustainable workflows to substitute conventional digital fabrication plastics for application areas that span wearables,^3–6^ packaging,^7,8^ and architectural structures.^9–14^ Yet the transition to more sustainable materials remains slow.

Following the quest for sustainable material alternatives, researchers have noted the potential of mycelium-bound biomass and adopted the technique of using biomass to cultivate mycelia to construct our built environment.^9,15–18^ Mycelium-based composites adopt an alternative fabrication approach that relies on the growth of fungal mycelium rather than extraction from finite resources. The formation of structural mycelium composites often involves mixing mycelium spawns and biomass as bulk substrates and shaping the mixture in a rigid mold.^17,19^ Under proper temperature and humidity conditions, the mycelium proliferates into a hyphal network, filling void spaces and binding the loose substrates.^20–22^ Unlike other compostable materials, mycelium biocomposites are mechanically tough.^21,23,24^ For example, a hemp-mycelium foam produced by Ecovative^23^ has comparable mechanical properties to expanded polystyrene foam^25^ and stands as viable substitute for packaging materials through mold forming.^7,12,19,26^ However, designing rigid molds for certain shapes—such as hollow structures or objects with intricate undercuts—presents considerable difficulty. Alternatively, researchers have explored constructing complex, self-supporting formworks using techniques like knitting, weaving, or a combination of rigid frameworks and soft fabrics^13,16,27–32^ to create pliable, organic molds. These soft molds are then filled with mycelium-laden biomass, binding and stiffening with the substrate as the mycelium grows, resulting in a solid, consistent structure. This method, however, posits new challenges such as uneven filling in the labyrinthine molds and distortion of soft molds, leading to inconsistencies in structural integrity.

3D printing addresses the aforementioned challenges associated with mold-based manufacturing. It eliminates the need to produce single-use molds for fabricating custom shapes in small batches and enables the fabrication of hollow structures. Recently, 3D printing technology has been applied to utilizing recycled and renewable materials as printing feedstock, such as algae,^10^ spent coffee grounds,^24,33,34^ spirulina,^35^ tea leaves,^36^ eggshell,^37^ and olive pomace,^38,39^ to create complex shapes and functional structures. Previous research into 3D-printed mycelium biocomposites has explored the creation of intricate structures using various biomass substrates, including cardboard,^9,40,41^ seashell,^42^ soil,^43^ clay,^44^ and agricultural by-products.^17,45,46^ These materials, formulated into extrudable biopastes, have proven to be suitable substrates for different strains of mushrooms. Unlike traditional mold-formed mycelium composites where the mycelium network penetrates throughout the loose substrate, the mycelium growth on 3D-printed substrates is primarily confined to the surface. This surface growth is due to the dense nature of the extruded substrate paste and further compaction during the printing process, which limits internal colonization.^47^ Similarly, incorporating coffee grounds into the substrate for mushroom growth has been a common practice in the mushroom cultivation community for small-scale production.^48^ We therefore hypothesize that there is a productive intersection between additive manufacturing and coffee grounds for producing mycelium biocomposites.

While some additive manufacturing systems have been proposed to deposit layers of spent coffee ground as mycelium substrate, they often suffer from limitations in formulation and complex instrumentation. These limitations arise from factors such as complex formulations of extruded material using mainly lab-grade chemicals and equipment^20,21^ or the low vitality of mycelium,^43^ resulting in insufficiently functionalized mycelium surfaces and crude geometries. In contrast to these constraints, our approach significantly simplifies the instrumentation with a lower barrier of entry. By utilizing spent coffee grounds as the primary component, our formulation stands out for its high coffee composition compared with previous works,^20,21,24,36^ maximizing the utilization and recycling of a readily available waste product. Additionally, spent coffee grounds are readily granulated and sanitized at high temperatures and pressures, offering a substrate requiring minimal pre-processing compared with other biomass formulations. In previous research that delved into 3D printing with living mycelium materials, ensuring the proper sanitation of the recycled substrate was shown to be crucial to the viability of mycelium, requiring excessive heat treatment to prevent microbial contamination before inoculating the desired mycelium species.^21,40^ Recent developments in substrate formulation have demonstrated successful cultivation of mycelium under non-sterile conditions, but the substrate needs to be formulated with lab-grade chemicals and the colonization of mycelium is insufficient.^20,49^ Our workflow enables the fabrication of mycelium-laden biocomposites in a non-sterile environment, simplifying the manufacturing process compared with existing methodologies.^20,21,40,49,50^ Besides enabling diverse form factors via 3D printing, researchers have also investigated the living properties of mycelium, allowing it to bind, fuse, and weld layered or separately manufactured parts.^22,42,50^ The exploration of using living biomaterial to create regenerative, self-adaptive, and growable structures has just begun.

Developing new materials requires low-level material tuning and process optimization steps,^51–53^ which is not always supported by off-the-shelf machines. Therefore, new materials often demand new hardware systems.^54,55^ To address this for our own material exploration, we developed a custom extrusion printhead that exposes key material properties as tweakable parameters. This extrusion printhead is easy to assemble, inexpensive yet functional, compatible with existing slicing software, and can be integrated into mainstream 3D printer control boards.

**FIG. 1.**
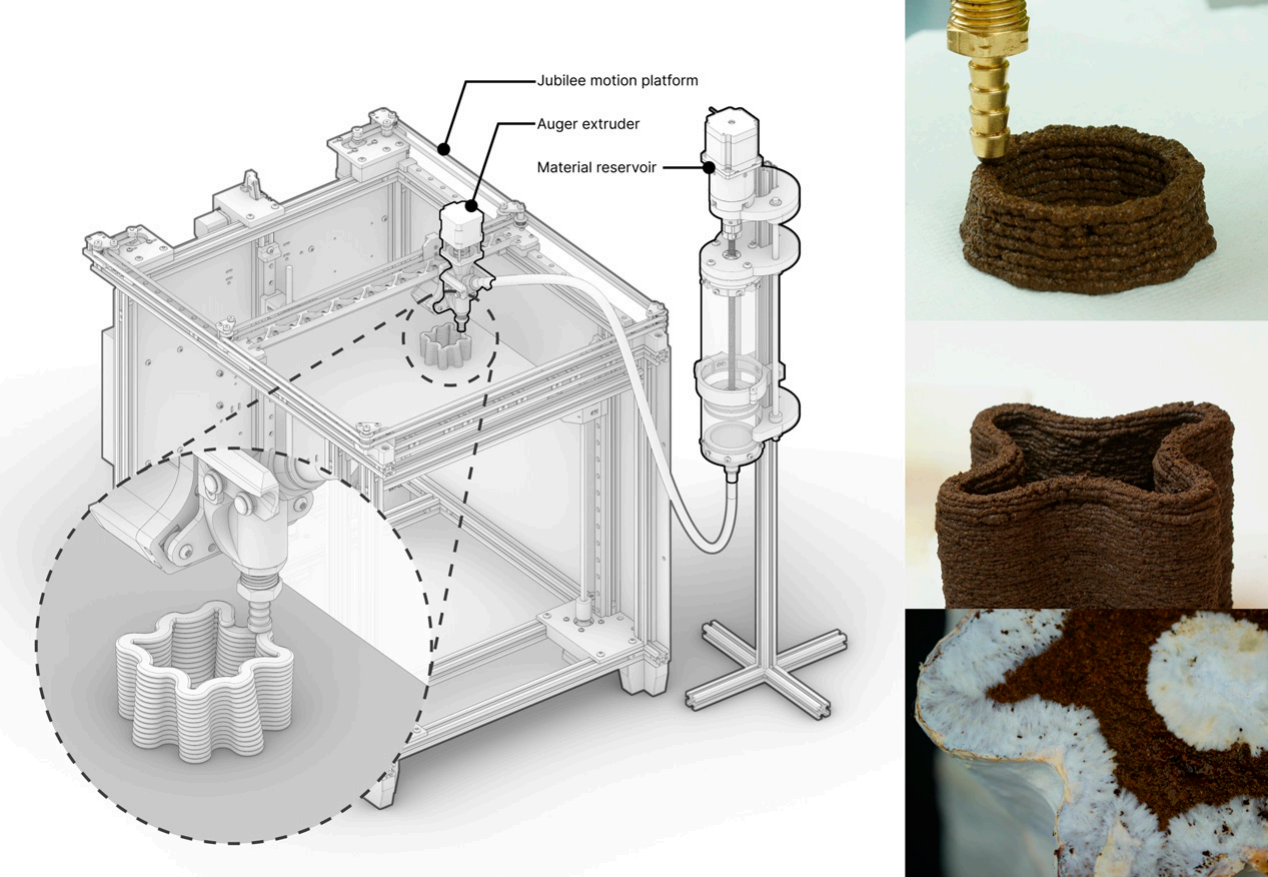
Overview of 3D-printed mycelium biocomposite. Our mycelium biocomposite paste, Mycofluid, is primarily composed of coffee grounds, can be 3D printed using a hobbyist 3D printer, and serves as the substrate for mycelium growth. Over time, mycelium colonizes the printed structure, bridging small gaps it encounters. This capability enables the creation of intricate geometries, including tall vases with delicate, thin walls.

In this work, we bring together the precision and customizability of additive manufacturing, using recycled materials and accessible tooling for small-scale customization, and the living nature of mycelium that enables bio-welding to create a sustainable fabrication process that prints custom and bespoke shapes. We demonstrate a workflow for 3D printing and growing mycelium biocomposite objects (Fig. 1) and highlight three contributions: *Fungibot*, a custom extrusion end-effector for biopaste; *Mycofluid*, a 3D-printable biopaste formulation with the highest constituent of spent coffee ground in the literature, accounting for 73% of the solid content (Table 1), inoculated with fungal spawns; and a method for cultivating mycelium in the 3D-printed structures (*Mycostructure*).

**Table 1.** Mycofluid Formula: Composition and Purpose of Each Ingredient

Ingredient	Weight %	Purpose
Spent coffee ground	54.9	Recycled material, spawning substrate, Mycofluid base
Brown rice flour	13.7	Providing carbohydrate
Ground grain spawn	5.5	Spawning substrate
Xanthan gum	1.1	Rheology modifier, homogenizer, binder
Water	24.7	Mycofluid base

## Materials and Methods

### Open-source biomaterial deposition system design

We present a system for 3D printing a unique material made of coffee grounds and binders. The 3D-printed structure acts as the substrate for mycelium inoculation. Our goal is to 3D print using large volumes (∼1 L) of sanitized and moisture-sensitive biopaste with variable viscosity. Off-the-shelf solutions are expensive (>$7k), difficult to interface with, and only accommodate a limited library of materials. We sought a low-cost open-source hardware alternative. Therefore, we ensured that all parts in our design were accessible off-the-shelf or by hobbyist-level computer numerical control (CNC) milling or 3D printing, i.e., *fabricatable.*^56,57^ Our total hardware cost is $200 for the extruder and $1500 for the motion platform, and the source and documentation are available at https://github.com/machineagency/fungibot and https://jubilee3d.com/.

#### Deposition system

**FIG. 2.**
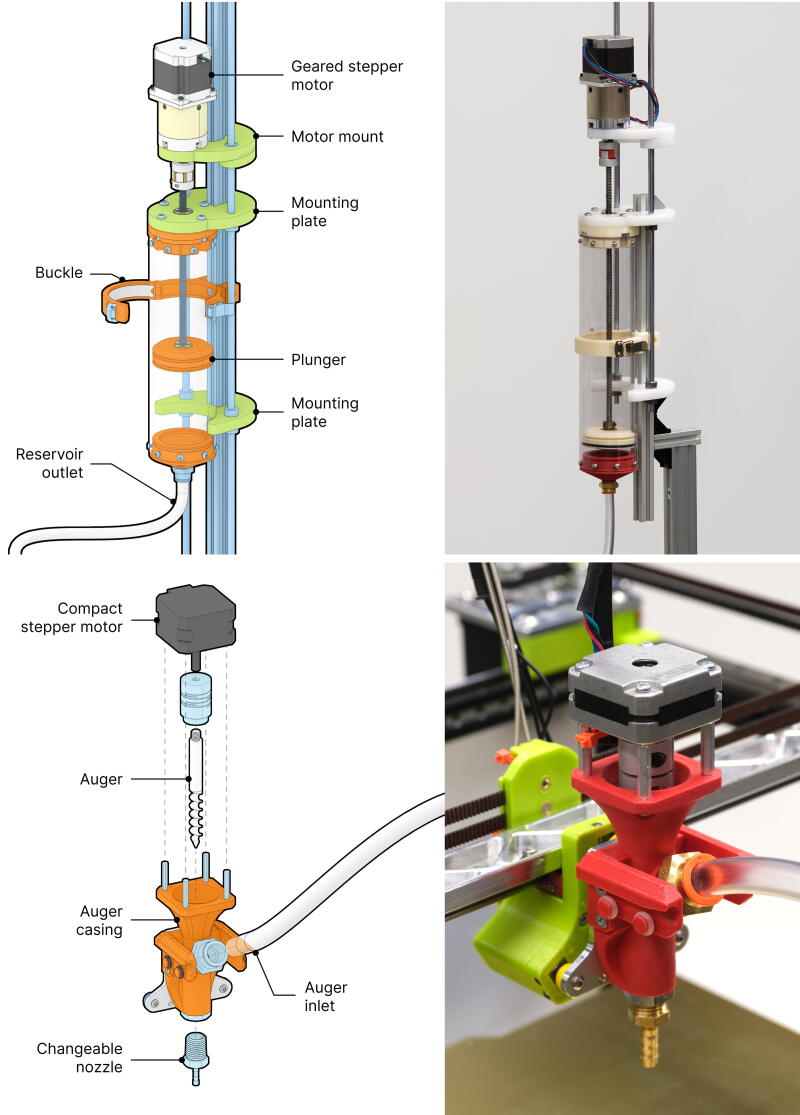
Fungibot consists of a material reservoir (*top*) that transports biopaste by a motor-driven plunger and an auger extruder (*bottom*) retrofitted on a desktop motion platform.

Fungibot comprises two main subsystems: the material reservoir and the printhead (Fig. 2). First, the material reservoir holds the biopaste and drives it through a flexible hose. Second, the printhead accumulates material from the flexible hose and controls its dispense onto a 3D printer build platform.

Having a large material reservoir reduces the time required for material preparation and minimizes batch variations. We use cut-to-size polycarbonate tube as the material reservoir; polycarbonate is clear, impact-resistant, and inexpensive. The polycarbonate tube has a 3-mm wall thickness, a 70-mm inner diameter, and is 300 mm long, accommodating up to 1 L per material reservoir. We selected this amount to be able to make artifacts of up to 300 × 300 × 300 mm, a typical build volume for higher-end hobbyist 3D printers.^56^ Additional material reservoirs can be easily stored (e.g., in a refrigerator) and swapped out.

Most 3D printing motion platforms are not engineered to bear heavy loads. One liter of our biopaste weighs over 1 kg, and the geared stepper motor alone weighs more than 1 kg. Drawing from existing open-source projects,^58–60^ we implemented a plunger-driven material reservoir in conjunction with a lightweight auger extrusion printhead, connected by a flexible hose. The material reservoir is capped at both ends, with one end connected to the auger extrusion printhead via tubing, while the other is mounted on an aluminum extrusion alongside two linear motion rods to ensure stability.

The material is pushed by a 3D-printed plunger and flows into the auger extrusion printhead (Fig. 2B). The auger is 8 mm in diameter. All artifacts shown were printed with a 5 mm nozzle to balance efficiency and aesthetics.

Both the plunger and the auger use stepper motors. There is a significant diameter reduction (70 mm to 5 mm) during printing, therefore substantial flow resistance. Hence, we use 15-fold gear reductions to increase plunger force, and a compact short-body auger motor.

### Mycofluid: a coffee-based mushroom-inoculated material for 3D printing

Here we describe our material formulation and mycelium growth method and the process that went into tuning the material properties and optimizing growing conditions. The formulation of coffee-based mycelium-laden *Mycofluid* is designed to satisfy three conditions:

1. Uniform granularity: maximizes coffee reuse while maintaining homogeneity;
2. Tuned viscosity: i.e., can be 3D printed;
3. Resource-efficient sterilization: uses minimal sterilizing methods.

The formulation of Mycofluid is shown in Table 1. Coffee grounds have a range of granular sizes contributing to different coffee drink styles. We used spent espresso coffee grounds because they are the finest, with an average grain size of 0.3 mm. Mycelium also flourishes with a supply of carbohydrates. Inspired by the PF-Tek (Psilocybe Fanaticus Technique), an easy and widely used indoor mushroom growing technique, we spiked the mycelium growth by adding brown rice flour. To make granular materials 3D printable, engineers often add rheology modifiers or binders to adjust the viscosity and homogenize the granular substrate. Mycofluid uses food-grade Xanthan gum (Modernist Pantry, ME) as a rheology modifier and binder. Finally, water is added to the recipe. Water changes the viscosity of Mycofluid and provides moisture for mycelium growth. Hence, as a new formula is developed, water is added at last gradually until the Mycofluid reaches a 3D-printable consistency.

### Assessing a material’s printability

To examine the printability of the coffee-based biocomposite, the flow behavior was determined for the formulation used to produce all samples in this work. The rheological tests were conducted using an Anton Paar MCR 301 Rheometer. Dynamic shear viscosity was measured over a range of shear rates from 0.1 to 2.5 s^−1^ with a 25 mm parallel plate and a 2 mm gap at ambient temperature and humidity. These test parameters were held constant across all samples and were selected to accommodate for the thick nature of the biocomposite. The results are shown in Figure 7A, which suggests a clear shear-thinning behavior.

**FIG. 3.**
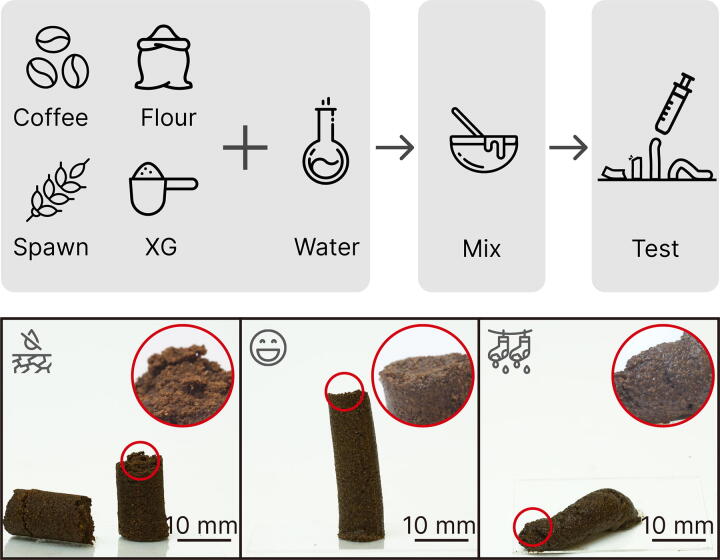
Method of assessing a material’s printability. We used a 10 mL syringe to visualize the printability of Mycofluid made of coffee grounds, brown rice flour, grain spawns, and Xanthan gum (XG). A 3D-printable paste should maintain a free-standing position with a slight tilt, indicating the right balance of elasticity and moisture content. Paste that is too dry may break during extrusion, while overly wet paste may not support itself during layering in 3D printing. Scale bar: 10 mm.

To enable rapid prototyping of a new material formulation, we derived a quick testing method^34,61^ as shown in Figure 3. The test uses a 10 mL syringe with graduated marks. First, trim the tip of the syringe and retain only the barrel. Second, load the syringe with 8 mL biopaste. Then, extrude the biopaste against a hard surface, such as a glass slide, retracting as the paste is deposited. Last, lift the syringe and leave the paste on the glass slide. A 3D-printable paste should remain in a free-standing position, slightly tilted, indicating proper elasticity and moisture content. A paste with a too dry consistency will likely break during the push and retraction. Dry paste exerts too much resistance during 3D printing and will not adhere to itself. If a paste is too wet, it will not be able to support its layers’ weight during printing.

### Mycelium cultivation

Mushroom cultivation requires sterilized conditions. We experimentally confirmed that as long as all the containers and surfaces are sanitized with alcohol wipes, contamination can be effectively prevented without the need for professional equipment such as laminar flow hoods. As described by mycologist Paul Stamets,^48^ the growth of mycelium follows three stages.

#### Spawn generation

Spawns are the vehicle of supplementation into a selective substrate. There are two primary methods for initiating mycelial growth within a substrate: mixing solid spawns and adding liquid spores. Solid spawns are typically grains inoculated with mushroom mycelium, while liquid spores are often packaged in sterile syringes. Liquid spore-mass inoculation is a common practice in mushroom farming on bulk substrates, but it requires a completely sterile environment. Commercially, grain spawns come in sterile containers, usually glass jars or plastic bags, and are preferred by home growers due to their ease of handling and resistance to contamination. We source grain spawn from a local supplier.

**FIG. 4.**
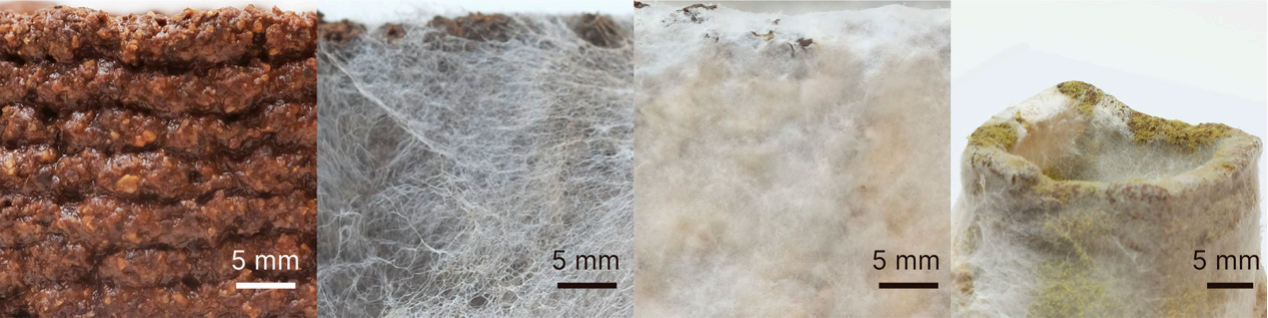
Progression of Mycofluid incubation. Left to right: freshly printed Mycofluid, 5 days of spawn run, 10 days of spawn run, improper sanitization, and weak spawns resulting in a contamination issue.

We tested both oyster mushroom grain spawns and Reishi grain spawns. We found that fully developed Reishi mushroom spawns demonstrated greater resistance to undesired microbial proliferation when subjected to the same operational procedures. While oyster mushroom spawns are a suitable choice for growing on coffee grounds, they are more susceptible to contamination in Mycofluid formulation, as illustrated in Figure 4.

#### Inoculation

Inoculation is the process of mixing spawns into a suitable medium to facilitate further mycelium growth. Home growers typically combine grain spawns with substrates of their choice, depending on material availability and the desired mushroom strain for cultivation. These substrates often consist of cost-effective recycled waste materials, such as sawdust and cardboard, and do not require sterilization due to their low nutritional value. This simplifies small-scale mushroom cultivation. Coffee is rich in nutrients, which could potentially make it susceptible to mold formation, yet spent coffee is relatively sterile due to the coffee-making process. We add ground Reishi (*Ganoderma lucidum*) grain spawns to inoculate spent coffee grounds.

#### Spawn run

During the spawn run, the mycelium colonizes the substrate. The growth of mycelium colonies depends on four factors: moisture, air exchange, temperature, and lighting. We maintain suitable conditions for the spawn run during incubation. The spent coffee grounds we sourced have a 45% water content, and the grain spawn has a 50% water content, resulting in a 52% water content for Mycofluid. It is recommended to maintain a water content between 60% and 75%.

### 3D-printing workflow

First, ensure that all containers, tools, and surfaces that will come into contact with the ingredients are thoroughly sanitized using 70% isopropyl alcohol. Gloves are optional; however, hands should also be sanitized if ungloved.

#### Step 1: mixing of ingredients

Measure all ingredients by weight (Table 1). Roughly mix all the dry and semi-dry ingredients using either hands or a suitable stirring tool. Add water to the mixture. Thoroughly blend the ingredients until the resulting paste achieves a uniform consistency. This prepared Mycofluid can be stored in a refrigerator for several days if covered.

#### Step 2: loading Mycofluid into Fungibot

Ensure that all inner surfaces of Fungibot, including the material reservoir, plunger, hose, auger, casing, and nozzle, are sanitized by thoroughly spraying with 70% isopropyl alcohol and then wiping with a clean paper towel to remove any residual alcohol. Secure the material reservoir onto the mounting plates and cap it. Advance the plunger motor until the material flows through the hose and enters the material inlet on the auger printhead. Be cautious to avoid the inclusion of large gas bubbles; small gas bubbles can be eliminated while printing by the auger mechanism.

#### Step 3: printing with Mycofluid

We design and adjust all 3D models using Rhinoceros (McNeel) and slice them using PrusaSlicer. All presets are available in Supplementary Data S1. Prior to sending machine commands, place a waterproof hardboard (such as a corrugated plastic sheet) large enough to accommodate the print on the printbed. Line it with a paper towel. Start printing by uploading the G-Code file through the web socket on a laptop computer connected to the Jubilee mainboard via ethernet. Detailed instructions on how to use the Jubilee motion platform can be found at https://jubilee3d.com/.

#### Step 4: colonization

Prints are transferred to a container with a lid. Additional moisture is provided by spraying the 3D-printed Mycofluid once a day. Fresh outside air is introduced when the lid is open. The container is placed in a thermostatic, low-light corner to further promote mycelium colonization. The spawn run for 3D-printed Mycofluid lasts for 10 days, as shown in Figure 4. On day 5, we observed loose but viable mycelium growth throughout the print, and by day 10, the print has been fully colonized with mycelium.

#### Step 5: drying

Fully colonized samples are air-dried at room temperature (22°C) for a minimum of 24 h. The ASTM D638 Type 4 dogbone samples are sandwiched between two drying meshes during this process to maintain their flat shape while drying.

#### Step 6: post-printing maintenance

All components of the 3D printer that came into contact with Mycofluid, including the used material reservoir, plunger, hose, auger, casing, nozzle, and print bed, are cleaned with soap and water.

### Performance

The structural integrity and physical properties of myco-composite were measured according to the following protocols. We make comparisons between 3D-printed coffee biocomposite and myco-coffee biocomposite to highlight the effect of mycelium on physical properties, mechanical properties, and water repellence.

#### Hydrophobicity, wettability, and absorbance

Hydrophobicity was assessed using static advancing contact angle measurements, adapted from ASTM D7334. A 0.5 mL droplet of distilled water was manually placed on the surface of each printed and colonized sample using a syringe, while video recording the process. The contact angle for each droplet was measured at 5-s intervals over a 60-s period. The Drop Shape Analysis plugin on ImageJ was used to measure the contact angle, and the results were averaged over three measurements.^62^

Water absorbance and swelling experiments were adapted from ASTM D1037. Three printed and three colonized samples, each approximately 30 × 30 × 30 mm^3^, were prepared. After being dehydrated overnight, the samples were submerged in room temperature water, fully covering the cubes. Volume and mass were measured for each sample before submersion and after blotting at 1, 2, and 24 h of submersion.

#### Mechanical testing

Tensile testing was conducted at ambient conditions using an AGS-X test frame from Shimadzu Scientific Instruments with a 500 N load cell. ASTM D638 Type 4 dogbone samples were prepared according to the described workflow. To evaluate the effect of mycelium colonization, we tested three to five samples of both uncolonized and colonized biocomposites. All samples were fully dried at room temperature before testing. The samples were strained at a rate of 1 mm/min until failure.

Compression testing was conducted using an AGS-X test frame from Shimadzu Scientific Instruments with a 500 kN load cell. The test samples were cubes with either 50% or 100% infill. The samples were compressed at a rate of 1 mm/min. For each parameter evaluated, three samples were included in the final analysis.

## Results and Discussion

**FIG. 5.**
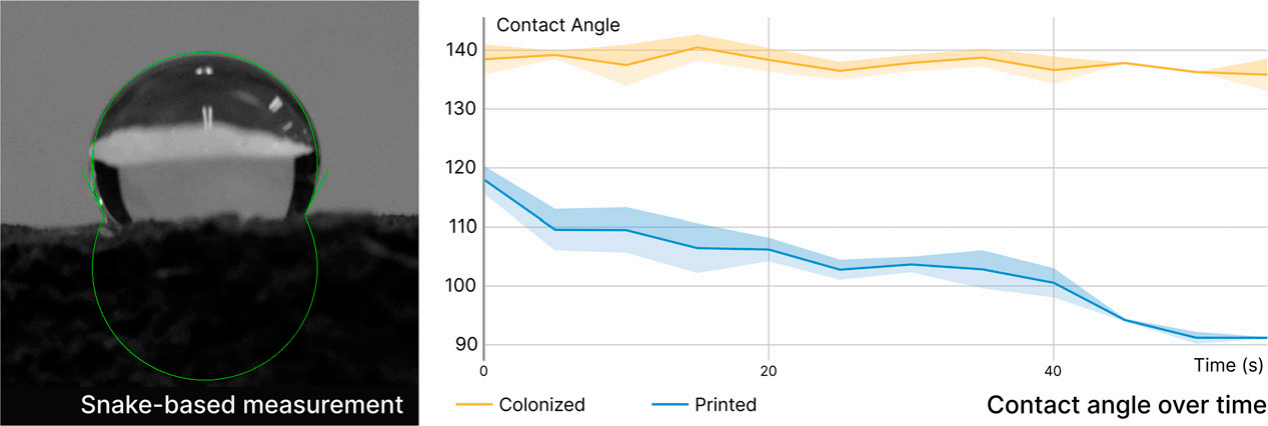
Contact angle change on mycelium-colonized and pristine biocomposite surfaces over time. **(A)** Droplet contact angle was measured over a period of 60 s at a 5-s interval to assess hydrophobicity. **(B)** Colonized biocomposite demonstrates better hydrophobicity over time than pristine coffee biocomposite.

**FIG. 6.**
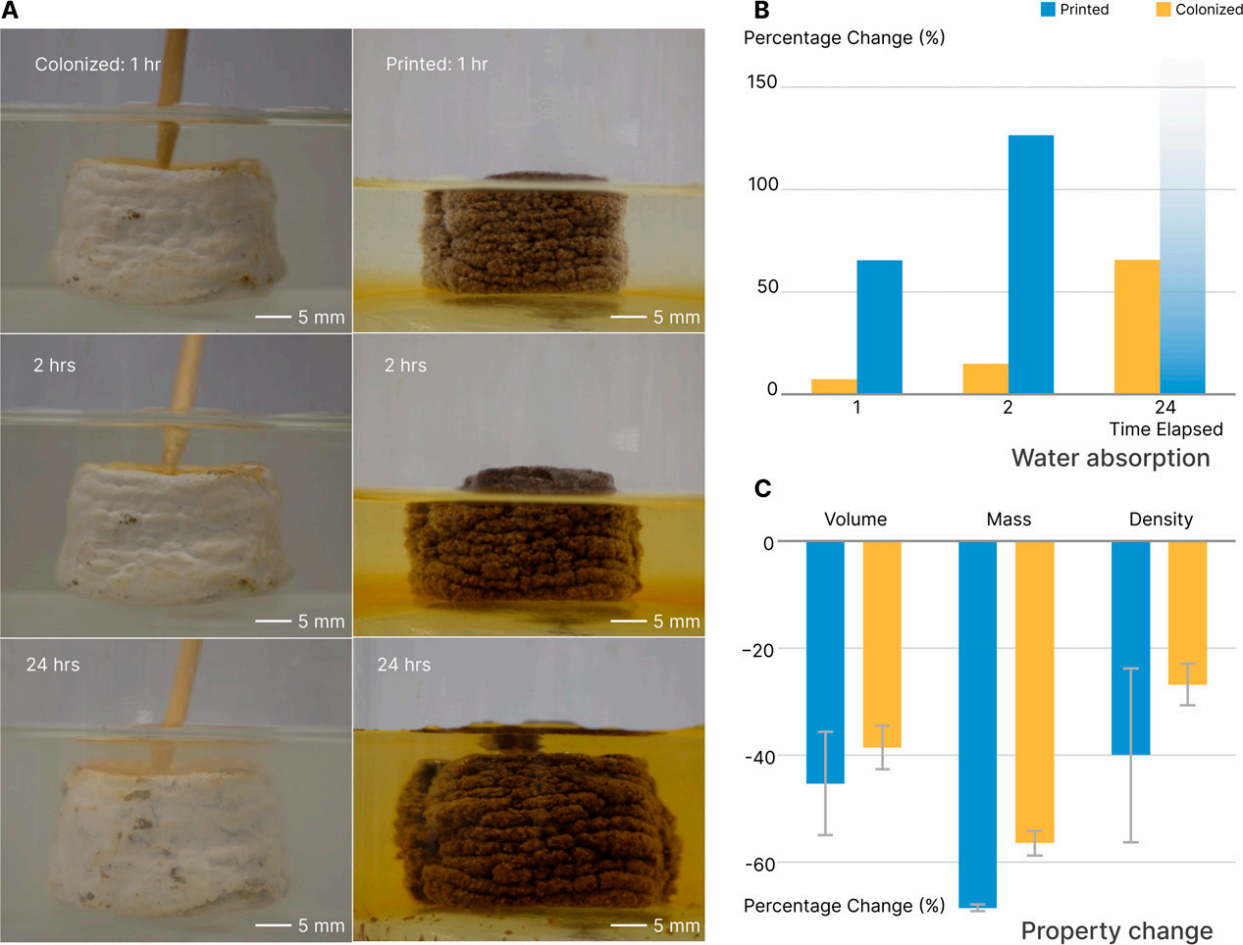
Property and morphological changes upon water absorption in printed and colonized coffee biocomposites. **(A)** Time series of colonized and uncolonized biocomposite cubes submerged in water. Note that after 24 h, the pristine coffee biocomposite disintegrated and could not be removed from the water. **(B)** Colonized biocomposites demonstrate resistance to water penetration after 24 h of submersion and can reversibly dry to restore their original weight and shape. **(C)** Colonized biocomposites exhibit smaller dimensional shrinkage and weight change upon drying.

Full colonization of coffee-based biocomposites forms a “skin” layer with enhanced hydrophobicity, indicated by a 138° contact angle (Fig. 5A). This mycelial skin also prevents water absorption over time. The contact angle on the mycelium skin remains stable, whereas the contact angle on the printed coffee-based biocomposite decreases from 118° to 91°, leading to a loss of hydrophobicity within 60 s. This water barrier effectively protects the encapsulated biocomposite, as evidenced by the weight change from 1-h water absorption, which decreases from 65% to 7%. Over time, pristine biocomposites fully dissolve in water (Fig. 6A), while colonized biocomposites only gain 66% in weight after 24 h. Additionally, the colonized biocomposites dry to within 5% of their original weight and maintain their shape.

**FIG. 7.**
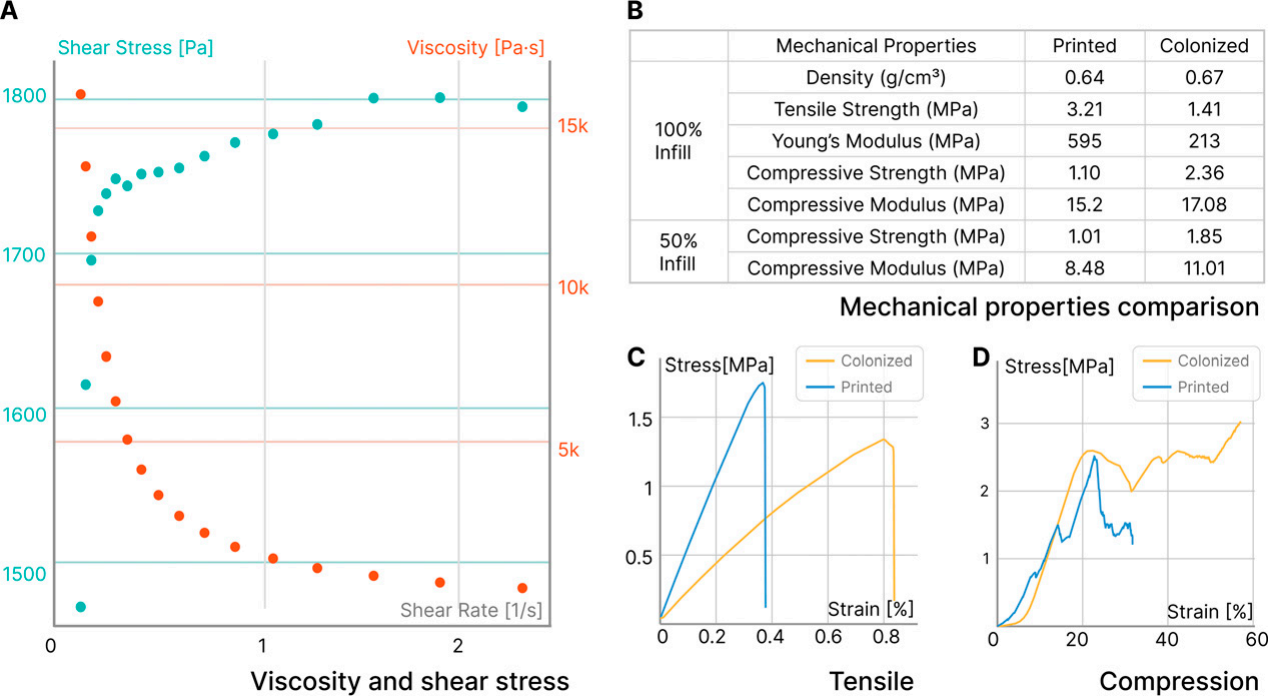
Rheological and mechanical performance of uncolonized and colonized coffee-based biocomposites. **(A)** The printing material exhibits clear shear-thinning behavior. **(B)** Mechanical properties of printed and colonized coffee-based biocomposites. Our formulation achieves the highest tensile strength (3.21 MPa) among similar uncolonized biocomposites. Upon colonization, tensile strength decreases to 1.41 MPa, while elongation at break doubles. **(C)** Colonized biocomposites demonstrate a continuous increase in compressive strength until buckling and yielding, whereas pristine coffee biocomposites show step-wise drops due to internal lattice failure.

The formation of the mycelial skin alters the physical properties of printed structures upon drying. The resulting mycelium composite is substantially denser than cast ones,^23^ likely due to the use of a rigid and compact substrate. Dimensional shrinkage and weight loss both decrease upon drying as the mycelial skin forms a ductile covering that prevents local cracking. In addition to causing cosmetic defects in pristine biocomposites due to shrinking, mechanical strength also changes. Our formulation achieves the highest tensile strength (3.21 MPa) among uncolonized biocomposites with similar formulations.^31,46^ After colonization, the tensile strength decreases to 1.41 MPa, while the elongation at break doubles from 0.4% to 0.8% (Fig. 7C). This is contrary to typical particulate-based mycelium composites, where mycelium serves as a binder and enhances mechanical properties. However, in our case, mycelium actively digests organic materials such as cellulose, which contribute to the rigid structure. The compressive modulus of the mycelium-colonized biocomposite is comparable to that of the pristine coffee biocomposite, likely due to the condensing effect caused by the formation of the mycelial skin. As shown in Figure 7D, the continuous rise in compressive strength for the colonized biocomposite indicates a larger area under the stress-strain curve, implying higher energy absorption before failure. This enhanced toughness is likely due to the mycelial network acting as a reinforcing matrix, distributing stress more evenly throughout the material and preventing catastrophic failure. In contrast, the step-wise drops observed in the pristine coffee biocomposite suggest brittle behavior with sudden releases of energy, characteristic of lower toughness caused by internal lattice failure. This difference in toughness between the two materials highlights the significant impact of mycelial colonization on the mechanical properties of the biocomposite. Additionally, we measured the tensile and compressive performance of pristine and colonized biocomposites with 50% infill. The mechanical performance only slightly falls short compared with solid biocomposites, suggesting the possibility of fabricating lighter yet still durable structures. These parameters indicate a tradeoff between mycelium growth and its associated functions and mechanical properties in this class of materials, which is an important consideration when designing materials for specific applications.

Mycelium-bound composite has not yet realized its promising potential in customized manufacturing and production using fully biodegradable and sustainable materials. We showcase how we harness the material properties of Mycofluid and living mycelium to produce unique Mycostructures with slightly different post-processing techniques.

### 3D printing with overhangs

**FIG. 8.**
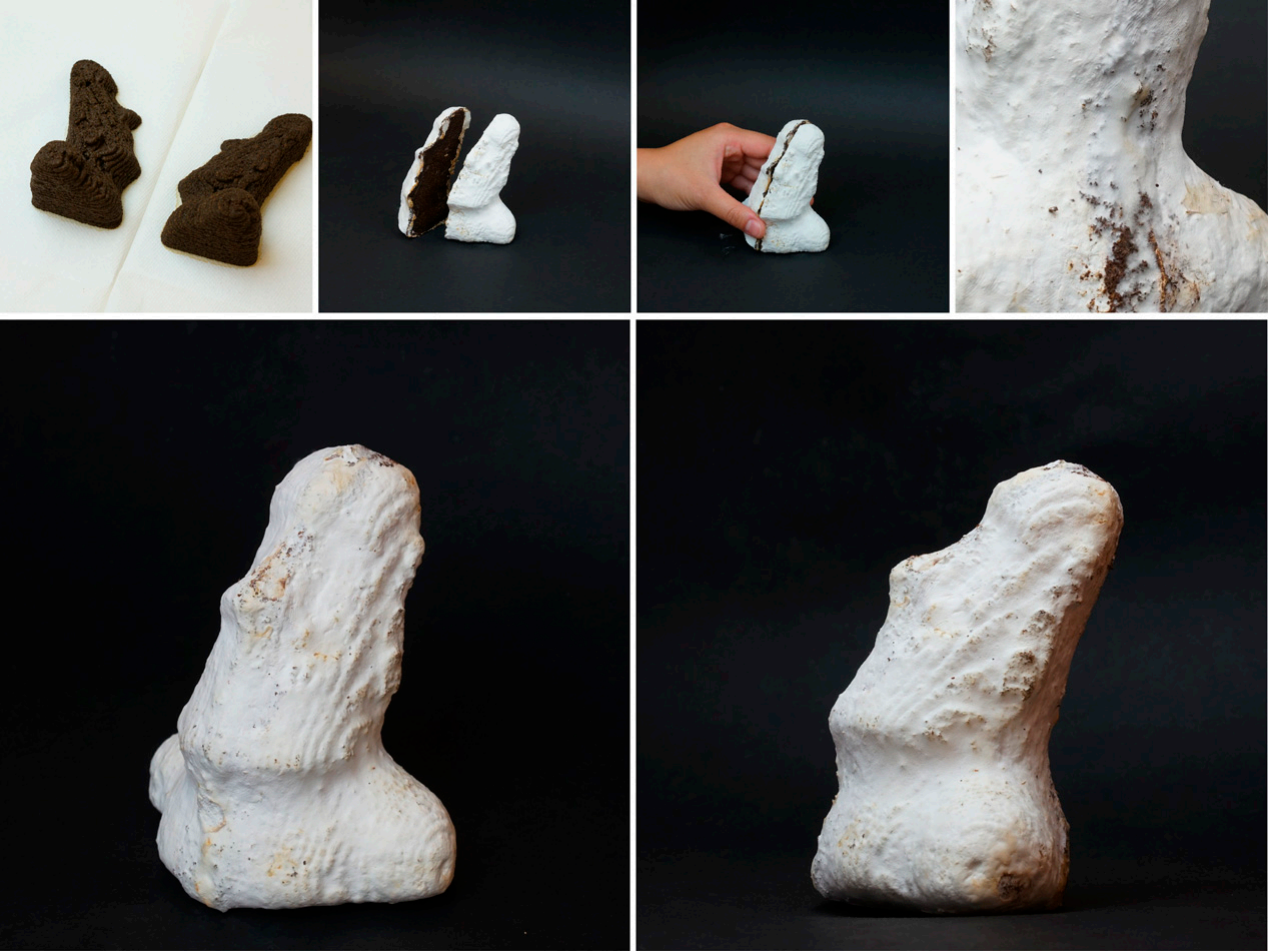
Illustration of a 3D-printed Moai statue, halved down the middle and laid flat for 3D printing. After the initial spawn run which lasted for a week, the outer surfaces of the statue became fully colonized by mycelium. We removed the prints from the incubation container, pressed the middle surfaces together, and then placed the fused one-piece back into the container. During a second incubation period, the mycelium continued to grow, effectively binding the two parts together.

3D printing overhangs and thin geometries with soft paste is challenging. Paste materials may not be as rigid or self-supporting as solid filaments, making them prone to deformation or sagging. In this example, we demonstrate how Mycofluid’s unique living property circumvents this challenge. A Moai statue 3D model^1^ has a neck above the shoulders and is difficult to print with soft materials. We halved the 3D model, printing two flat sides. After a 1-week initial spawn run, mycelium fully colonized the outer surfaces. The prints were fused together by mycelium during a subsequent incubation period, resulting in a solid single piece (Fig. 8). The statue was left to dry in the air.

### 3D printing with tall walls

**FIG. 9.**
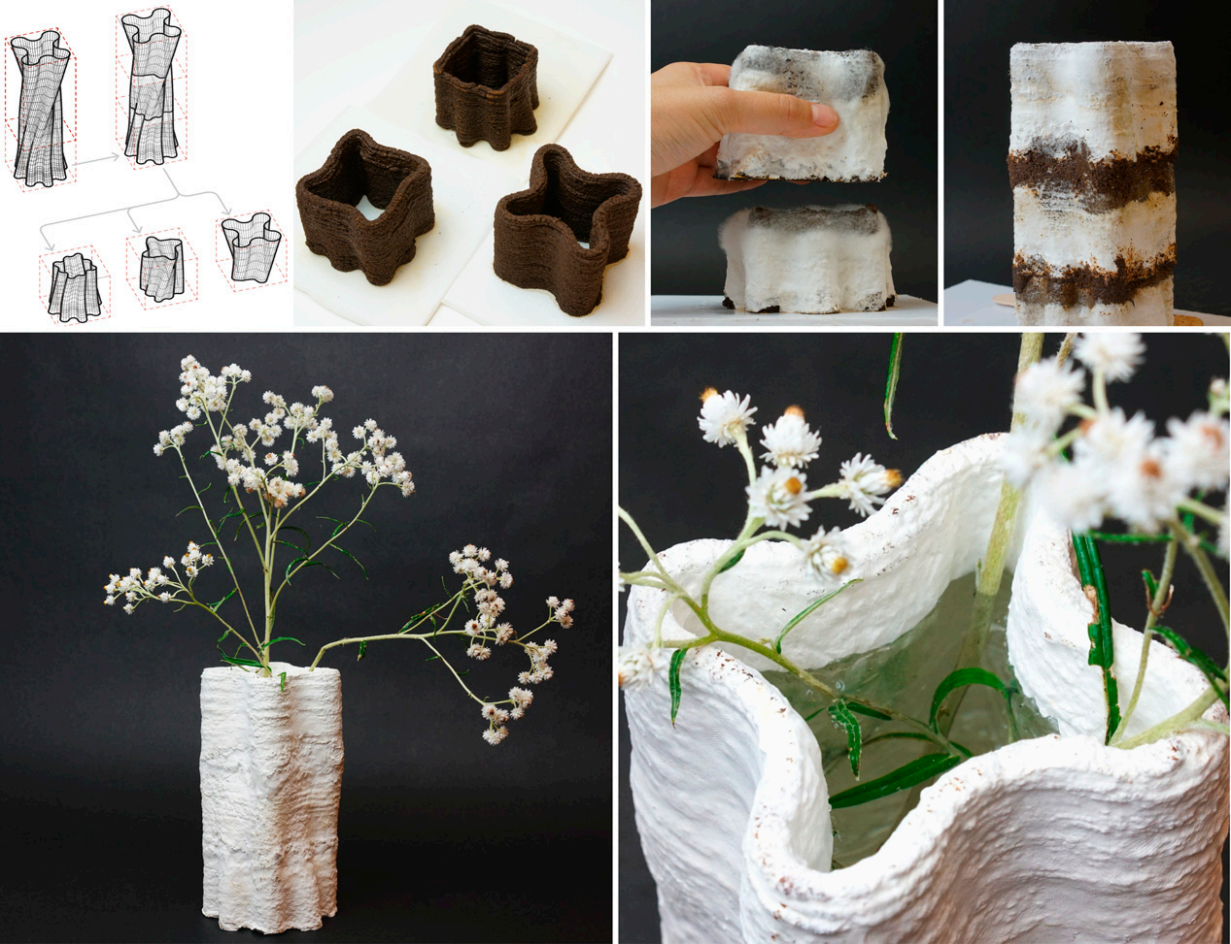
Illustration of a tall, freeform vase 3D printed as three equal-height pieces. The 3D model of the vase was prepared in Rhinoceros first. All three pieces were sliced using Vase Mode in PrusaSlicer, with only the bottom piece printed using a single layer for the bottom. After a 1-week spawn run, the vase pieces were stacked and fused together by mycelium, resulting in a fully colonized, single-piece structure with added waterproofing properties.

Tall structures with thin walls are difficult to 3D print with soft materials due to the lack of self-supporting capability required to sustain their own weight. In this demonstration (Fig. 9), we designed a tall vase and divided it into three equal-height pieces for 3D printing. Following a 1-week spawn run, we stacked the three pieces together and applied a thin layer of Mycofluid at the interfaces. Then, the stacked vase was placed back for the fusing incubation process. Once fully colonized and dried in the air, the vase transformed into a single, structurally sound piece. Additionally, the mycelium added waterproofing properties to the vase.

### 3D printing with hollow structures

**FIG. 10.**
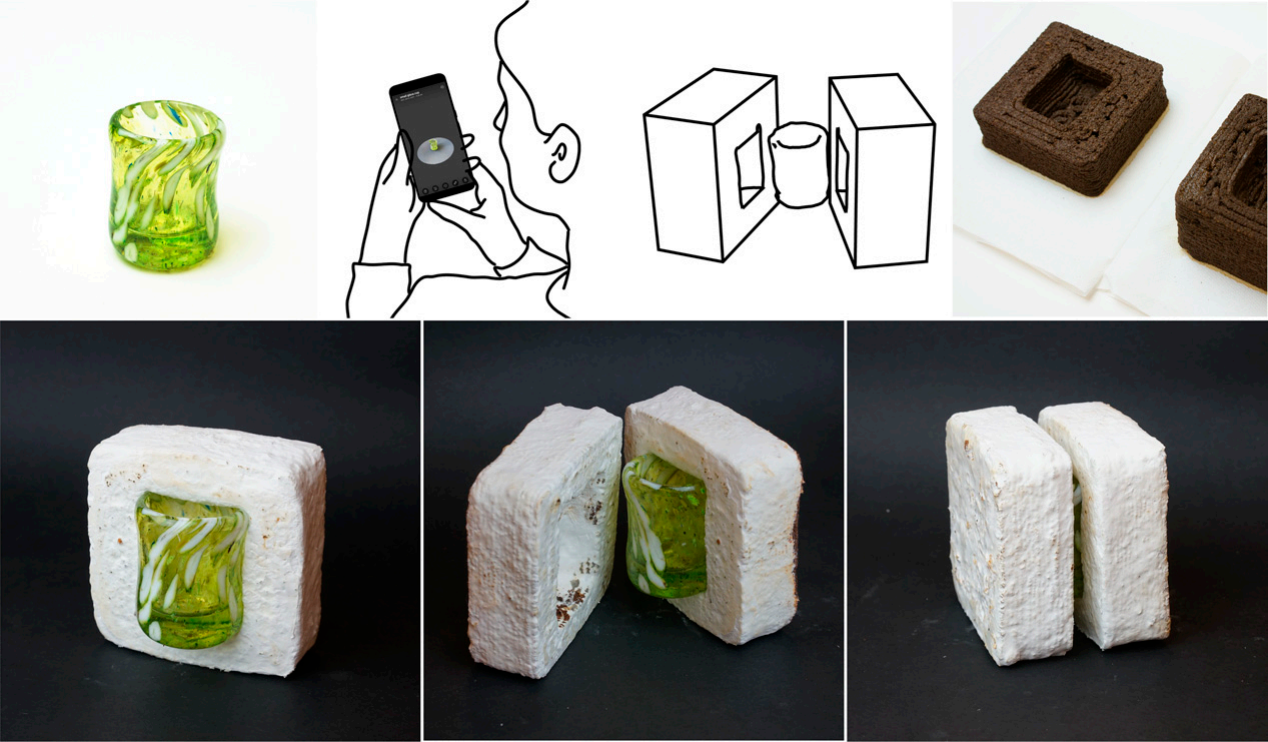
Illustrating the creation process of a two-part packaging material designed for safeguarding a delicate glass cup. The process starts with scanning the cup using Scaniverse, a 3D Scanner app, on a mobile device. Subsequently, the 3D model of the cup is edited in Rhinoceros to produce a two-part mold that envelops it. The two-part mold is then 3D printed using Mycofluid and incubated for a period of 10 days, resulting in a snug-fitting packaging material for the cup. The resulting packaging material provides a snug fit for the original object.

Mycostructures are water-repellent and lightweight once dried, making them a suitable substitute for Styrofoam as packaging materials. 3D printing allows small-scale production of customized packaging for fragile shipments. We demonstrate a workflow in which we customized a shipping packaging material for a unique, non-uniform object, as shown in Figure 10. The process started with scanning the object, a handmade cup, using Scaniverse, a 3D scanner app, on a mobile device. Subsequently, the 3D model of the cup was edited in Rhinoceros to produce a two-part mold that envelops it. The mold was then 3D printed using Mycofluid and incubated for a period of 10 days, resulting in a snug-fitting packaging material for the cup.

### 3D printing with biodegradable material

**FIG. 11.**
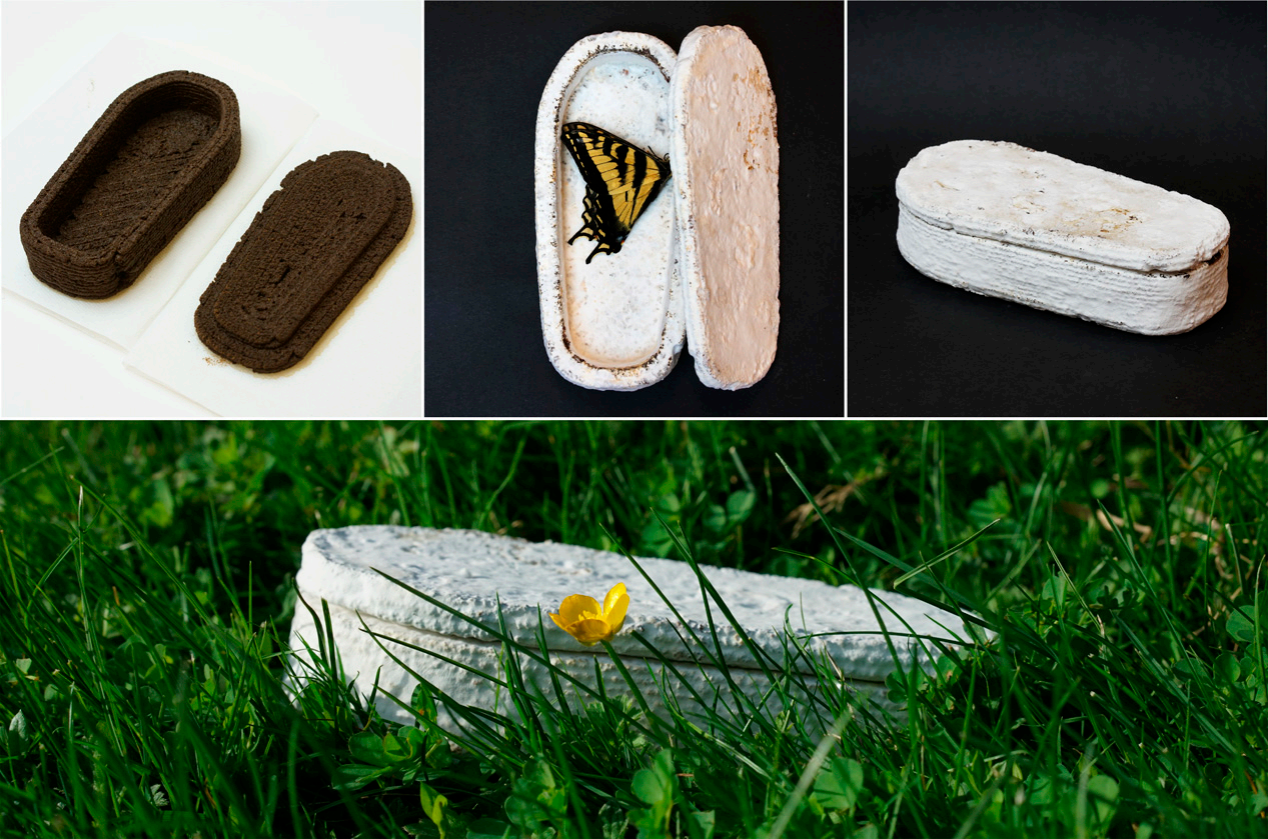
Illustration of a two-part coffin. The parts are printed with Mycofluid separately and incubated for 10 days. Shown is a swallowtail butterfly, found dead by the authors. We placed it in the coffin and closed the lid. The coffin will fuse and decompose with the insect underground.

Finally, we want to highlight the potential of Mycostructure for biodegradable applications. Inspired by Bob Hendrikx’s Living Coffin,^63^ we made a mini-coffin with a removable lid, as shown in Figure 11.^2^ Note that the Reishi mushroom (*G. lucidum*) used in this study is only a wood-decaying species, this method validates the possibility of developing such biocomposites with active purifying power.

Our methods implementation reflects design choices that come with trade-offs and limitations.

### Sterility must be maintained throughout the 3D-printing process and during the spawn run

The conditions suitable for cultivating Reishi fungi are also amenable to the growth of unwanted molds. Consequently, it is necessary to establish a clean workspace for the printer, devoid of airborne debris. We argue that our setup, combined with the sterilization precautions mentioned earlier, remains considerably simpler than a clean room.

### The mycelium on the bottom layer also needs air exchange for growth

Mycelium will not colonize the underside of prints until they are detached from the base sheet and repositioned. This requires careful manual handling by the operator. However, this is not always applicable when the bottom layer of a print is being fused, as demonstrated in some examples in “Results” section. Moreover, during incubation, Mycofluid retains its malleability. While prints can be handled by hand, the operator should manipulate prints with caution.

### Printing quality is influenced by both the material and the machine

The grainy ingredients in Mycofluid, despite rheological tuning, still limit the creation of objects with fine features. Mycostructures are robust, but we did not formally examine the mechanical properties of the objects. Like other extrusion-based 3D printers, Fungibot is an open-loop system. As a result, achieving reliable stopping and starting of the extrusion can be challenging—there are no sensors to detect issues such as clogs, excessive material flow, or other factors that might affect the final print. These issues can subsequently impact print quality and feature size.

### Compostability is unverified

Mycofluid comprises fully compostable ingredients. While this study did not include a formal compostability test, previous research has indicated that mycelium can inoculate and decompose coffee grounds, grain spawns, and brown rice flour.^48^

### Fabrication is slow

As we rely on the speed of mycelial growth, Mycostructures require over a week to make. However, the resulting artifacts are robust and sustainable.

## Conclusion and Future Work

This work presents a method for fabricating mycelium biocomposites. Our method relies on custom 3D printing hardware, the Fungibot, a biopaste optimized for 3D printing, Mycofluid, and an incubation protocol to care for the mycelium growing on the 3D-printed substrate. The resulting Mycostructures have several benefits: they are hydrophobic, unlike many other compostable materials, they do not rely on molding for fabrication, and they can self-fuse large structures from smaller parts. We argue that current research has only scratched the surface of what is possible with living materials; this article demonstrates that 3D printing with fungi is one promising direction. Using the workflow presented in this work, it is also possible to expand the library of mycelium-laden materials with accessible, local, and recycled biomass other than spent coffee grounds.

The primary contribution of this work is the development and accessibility of living biomaterials as building blocks. With proper tooling, this framework of creating living artifacts has the potential to be the new manufacturing paradigm in construction, repair, and maintenance on a large and continuously expanding scale using solely sustainable and recyclable materials.

## Notes

^1^ Moai statue by Julien-DaCosta: https://cults3d.com/:13676

^2^
The butterfly was found dead. No animals were harmed in creating this work.
